# Unmasking Semaglutide-Induced Gastroparesis: The Dangers of Rapid Dose Escalation in a Diabetic Patient

**DOI:** 10.7759/cureus.91679

**Published:** 2025-09-05

**Authors:** Rohan Singhal, Dheerja Sachdeva, Kevin Wortman II, Rekha Lall

**Affiliations:** 1 Medicine, Atal Bihari Vajpayee Institute of Medical Sciences and Dr. Ram Manohar Lohia Hospital, New Delhi, IND; 2 Medicine, Hamdard Institute of Medical Sciences and Research, New Delhi, IND; 3 Nephrology, Tulane University School of Medicine, New Orleans, USA; 4 Internal Medicine, Edward Via College of Osteopathic Medicine, Auburn, USA

**Keywords:** diabetes mellitus type 2, gastric emptying, glp-1 receptor agonist, ozempic gastroparesis, semaglutide

## Abstract

Gastroparesis is a chronic disorder characterized by delayed gastric emptying without mechanical obstruction, causing symptoms such as nausea, vomiting, and abdominal discomfort. Semaglutide, a glucagon-like peptide-1 (GLP-1) receptor agonist used for type 2 diabetes, can slow gastric motility and induce gastroparesis. We present a case of a 48-year-old woman with type 2 diabetes, who developed persistent nausea and vomiting after resuming semaglutide at 2 mg subcutaneously once weekly without following the recommended stepwise titration schedule (typically initiated at 0.25 mg weekly with gradual escalation). Shortly thereafter, she developed acute kidney injury secondary to dehydration. Imaging also demonstrated left-sided colitis, for which she received antibiotic therapy. Gastroparesis was suspected due to semaglutide use. Treatment with metoclopramide and cessation of semaglutide resulted in symptom resolution and recovery. Thus, semaglutide-induced gastroparesis should be considered in diabetic patients presenting with gastrointestinal symptoms, especially with rapid dose escalation. Early recognition and discontinuation of the drug can lead to swift symptom resolution and prevent complications.

## Introduction

Gastroparesis is a chronic disorder characterized by delayed gastric emptying in the absence of mechanical obstruction. This condition manifests with a spectrum of symptoms, including nausea, vomiting, early satiety, bloating, and abdominal discomfort [1]. The pathophysiology of gastroparesis involves impaired motility of the stomach, resulting from dysfunction of the autonomic nerves, smooth muscle abnormalities, or disruptions in the enteric nervous system [2]. Common etiologies include diabetes mellitus (particularly longstanding or poorly controlled), postsurgical complications, viral infections, and, increasingly, the use of medications that affect gastrointestinal motility. In a significant proportion of cases, the underlying cause remains unknown (idiopathic) [3].

Medication-induced gastroparesis is becoming more well acknowledged, especially as glucagon-like peptide-1 (GLP-1) receptor agonists are used more frequently.

GLP-1 is an incretin hormone secreted from intestinal L-cells in response to nutrient intake. It stimulates glucose-dependent insulin secretion, suppresses glucagon release, slows gastric emptying, and promotes satiety through central and peripheral pathways. GLP-1 receptor agonists replicate these effects, making them effective agents for glycemic control and weight management. Their mechanism involves enhanced insulin release, delayed gastric emptying through reduced antral and duodenal motility with increased pyloric tone, and appetite suppression mediated via central nervous system pathways [4]. GLP-1 receptor agonists, such as semaglutide, marketed as Ozempic (subcutaneous, once weekly) and Rybelsus (oral, once daily) for type 2 diabetes mellitus, and as Wegovy (subcutaneous, once weekly) for obesity irrespective of diabetes status, have become a cornerstone therapy owing to their efficacy in improving glycemic control and promoting weight loss [5]. These agents mimic the effects of endogenous GLP-1, enhancing glucose-dependent insulin secretion, suppressing glucagon release, and notably slowing gastric emptying. The latter effect is mediated through both central and peripheral pathways, including direct inhibition of antral and duodenal motility and increased pyloric tone, which collectively contribute to delayed gastric transit [6]. While this mechanism underlies their metabolic benefits, it also predisposes patients to gastrointestinal side effects such as nausea, vomiting, and abdominal pain, which are among the most frequently reported adverse events with these medications [7].

Recent studies and case reports have highlighted an increased risk of gastroparesis associated with GLP-1 receptor agonist use, particularly semaglutide [8-10]. Although most gastrointestinal symptoms are mild and transient, some patients develop persistent and severe gastroparesis, necessitating clinical recognition and intervention. The diagnosis of drug-induced gastroparesis requires demonstration of delayed gastric emptying in the absence of mechanical obstruction, typically via gastric emptying studies or endoscopic findings. Importantly, symptoms of gastroparesis often improve or resolve after discontinuation of the offending agent, underscoring the need for early identification and management [1].

It is vital to recognize semaglutide-induced gastroparesis since symptoms typically subside when the offending substance is quickly identified and discontinued. In this case study, a patient who experienced symptomatic gastroparesis after semaglutide therapy is highlighted, underscoring the significance of prompt awareness and treatment for patients who present with new-onset gastrointestinal symptoms while receiving GLP-1 receptor agonist therapy.

## Case presentation

A 48-year-old female with a history of type 2 diabetes, hypertension, gastroesophageal reflux with a mild stricture and small hiatal hernia, and chronic constipation presented to the emergency department complaining of nausea and non-bilious, non-bloody emesis for approximately three to four weeks. She was on 2 mg semaglutide subcutaneously once weekly for diabetic management and had stopped taking it for one month. After visiting her primary care physician, she was advised to restart semaglutide at 0.5 mg subcutaneously once weekly and then gradually titrate the dose upward until she reached her previous maintenance dose. However, she restarted treatment directly at 2 mg subcutaneously once weekly without re-titration and continued to take this for the past four weeks. She also reported left lower quadrant abdominal pain, subjective fevers, chills, and constipation. She denied any recent sick contacts, hematemesis, hematochezia, melena, and changes in urinary habits. Her home medications included semaglutide, metformin, lisinopril, hydrochlorothiazide, sucralfate, omeprazole, and docusate as needed.

In the emergency department, she was afebrile with a temperature of 97.7°F, heart rate of 84 beats per minute, respiratory rate of 20 breaths per minute, blood pressure of 106/74, saturating at 100% on room air, with a body mass index of 32.45. On exam, she was alert and oriented to person, place, and time, and not in cardiopulmonary distress. Heart sounds were at a regular rate and rhythm. Lungs were clear to auscultation bilaterally. The abdomen was soft, not distended, with normoactive bowel sounds. There was tenderness to palpation in the left greater than the right lower quadrants. She had no edema on her lower extremities, and her neurologic exam was nonfocal. Pertinent laboratory investigations have been summarized in Table 1.

**Table 1:** Laboratory investigations. GFR: glomerular filtration rate; AKI: acute kidney injury; LLQ: left lower quadrant; RLQ: right lower quadrant.

Category	Investigation	Result/findings	Reference range
Vital signs	Temperature	97.7°F (afebrile)	97°F - 99°F
	Heart rate	84 bpm	60 - 100 bpm
	Respiratory rate	20 breaths per minute	12 - 20 breaths/min
	Blood pressure	106/74 mmHg	90/60 - 120/80 mmHg
	Oxygen saturation	100% on room air	≥95%
	Body mass index	32.45 (obese range)	18.5 - 24.9
Physical examination	Abdominal exam	Soft, non-distended, normoactive bowel sounds; LLQ > RLQ tenderness	
	Cardiopulmonary exam	Normal heart sounds; lungs clear bilaterally	
	Neurologic exam	Nonfocal	Normal: no deficits
	Extremities	No edema	Normal: no swelling
Laboratory tests	Potassium	3.7 mmol/L	3.5 - 5.1 mmol/L
	Chloride	102 mmol/L	98 - 107 mmol/L
	Blood glucose	83 mg/dL	70 - 100 mg/dL (fasting)
	Serum creatinine	2.1 mg/dL (elevated; AKI)	0.6 - 1.3 mg/dL
	Baseline creatinine	0.8 mg/dL	0.6 - 1.3 mg/dL
	GFR	32 mL/min	>90 mL/min (normal)
	White blood cell count	12.8 x10³/µL (elevated)	4.0 - 10.0 x10³/µL
	Hemoglobin A1c	6.3%	<5.7% (normal); <7% (diabetic target)
	Urinalysis	Trace leukocyte esterase, 1+ bacteria, no WBCs or RBCs	
Imaging	CT abdomen/pelvis (non-contrast)	Uncomplicated left-sided colitis	
Microbiology & stool	Blood cultures	Negative	
	Urine culture	Negative	
	Stool culture	Negative	
	Fecal lactoferrin	Negative	
	Stool calprotectin	55 mcg/g (mildly elevated)	<50 mcg/g

**Figure 1:**
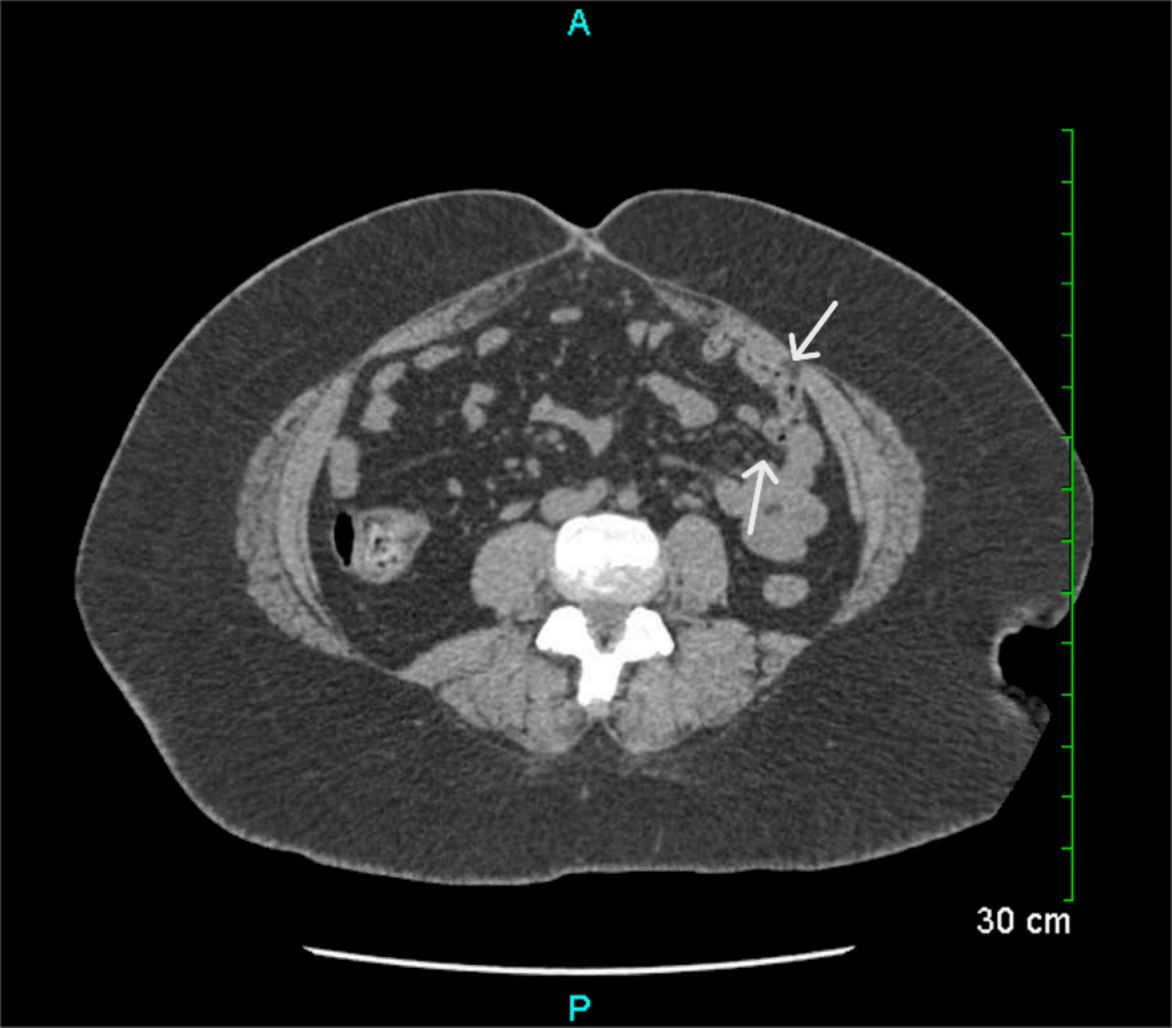
Non-contrast CT of the abdomen and pelvis showing multiple scattered diverticula. The arrows highlight an enlarged diverticulum in the sigmoid colon with surrounding mild fat stranding, consistent with mild, uncomplicated diverticulitis.

Non-contrast CT of the abdomen and pelvis revealed multiple scattered diverticula and associated pericolic fat stranding, consistent with uncomplicated diverticulitis (Figure 1). She was admitted to the hospital for intractable nausea/vomiting as well as treatment of her left-sided colitis.

She was initially started on ceftriaxone and metronidazole for the treatment of her colitis and potential UTI. Her nausea and vomiting were suspected to be semaglutide-induced gastroparesis. She was started on 10 mg metoclopramide as needed every six hours and maintenance fluid. After three doses of metoclopramide, her nausea and vomiting resolved. Her acute kidney injury resolved on her second day of hospitalization, after receiving 2 L of fluids. Blood, urine, and stool cultures returned negative on day four of her hospitalization. Fecal lactoferrin was negative, but stool calprotectin was marginally elevated to 55 mcg/g, suspected to represent inflammation from her colitis. Her abdominal pain appeared to be resolving. She was discharged after four days of hospitalization. She was prescribed a 10-day course of ciprofloxacin and metronidazole for her colitis and advised to follow up with her primary care provider regarding her diabetic medication regimen.

## Discussion

This case highlights the association between semaglutide and symptomatic gastroparesis, particularly in patients with pre-existing gastrointestinal (GI) comorbidities. In this patient, semaglutide was restarted directly at 2 mg subcutaneously once weekly, the maximum approved dose for type 2 diabetes, without adherence to the recommended titration schedule. The absence of gradual dose escalation likely amplified the drug’s inhibitory effects on gastric motility, precipitating severe nausea, vomiting, and subsequent acute kidney injury due to dehydration.

Furthermore, this report adds to the emerging body of evidence linking semaglutide use with the development of clinically significant gastroparesis. It underscores the importance of clinical vigilance, appropriate dose titration, and prompt recognition of gastrointestinal intolerance in patients treated with GLP-1 receptor agonists [8]. While semaglutide provides substantial benefits in glycemic control and weight reduction, clinicians must remain aware of its potential to induce persistent and severe gastrointestinal adverse effects consistent with gastroparesis.

Semaglutide’s agonism of GLP-1 receptors directly slows gastric motility by inhibiting postprandial antral contractions and accelerating pyloric pressure waves, creating a functional obstruction [9]. While this mechanism aids glycemic control and weight loss, it predisposes patients, especially those with GERD, hiatal hernias, or chronic constipation, to gastroparesis.

Notably, 19% of patients on GLP-1 agonists develop delayed gastric emptying, with semaglutide posing a 3.3-fold higher gastroparesis risk than bupropion-naltrexone and 6.1-fold higher than sleeve gastrectomy [9].

A review of recent literature reveals a consistent pattern: patients on semaglutide present with nausea, vomiting, abdominal pain, or early satiety, often in the absence of other identifiable causes. For example, Chaudhry et al. [11] reported a case of a 53-year-old woman with obesity who developed classic gastroparesis symptoms after four months on semaglutide. Endoscopy revealed retained gastric contents persisting for over 24 hours, and her symptoms resolved promptly after discontinuing the medication. Similarly, Kalas et al. [12] described two diabetic patients who were initially misdiagnosed with diabetic gastroparesis due to overlapping symptoms like postprandial pain, bloating, and fullness. In both instances, mechanical obstruction and other etiologies were excluded, and a detailed medication review revealed recent initiation of semaglutide. Gastric emptying studies confirmed delayed motility, and both patients experienced complete symptom resolution and normalization of gastric emptying after stopping semaglutide. This highlights the diagnostic challenge in differentiating medication-induced from diabetic gastroparesis, especially given shared risk factors, and underscores the importance of reviewing medication history and symptom timing. The authors emphasize that timely cessation of the offending agent typically leads to clinical improvement, mirroring the course observed in our patient. Additionally, Gomez et al. [13] presented a case involving a patient with long-standing type 2 diabetes who developed persistent nausea, vomiting, and abdominal pain soon after starting semaglutide. Despite dietary and pharmacologic interventions, the patient’s symptoms persisted until semaglutide was discontinued, after which both symptoms and gastric emptying normalized within a month. This case closely parallels our own, reinforcing the need for clinicians to recognize semaglutide as a potential cause of gastroparesis, particularly in diabetic patients, and to promptly withdraw the medication to facilitate recovery and avoid unnecessary interventions.

Management of semaglutide-induced gastroparesis centers on immediate discontinuation of the medication, as symptom resolution typically occurs within four to eight weeks following cessation. Aggressive hydration is crucial, especially when complications such as prerenal acute kidney injury arise from persistent vomiting and poor oral intake. In this patient, creatinine rapidly normalized after intravenous fluids, underscoring the importance of prompt volume repletion [11]. In cases where symptoms are severe or do not resolve immediately after drug cessation, prokinetic agents such as metoclopramide may be considered to accelerate gastric emptying and provide symptomatic relief [14]. Metoclopramide, a dopamine antagonist, is commonly used as a first-line pharmacologic treatment for gastroparesis and can be particularly helpful during the transition period after stopping the GLP-1 receptor agonist [15]. Dietary modifications, specifically consuming small, low-fat meals and avoiding eating close to bedtime, can help mitigate symptoms like bloating and reflux during recovery.

Nevertheless, a subset of patients may experience persistent gastrointestinal symptoms that require long-term gastroenterology follow-up and individualized care. This variability highlights the importance of personalized risk stratification, particularly in patients with pre-existing strictures or baseline motility disorders, who may be at higher risk for chronic complications.

Clinically, this case underscores the necessity of strict adherence to semaglutide titration protocols, such as restarting at 0.25 mg per week and escalating slowly, to minimize gastrointestinal adverse effects [16]. Providers should consider baseline gastric emptying studies in high-risk patients and educate all patients to promptly report new or worsening GI symptoms to prevent complications like acute kidney injury or malnutrition. Future research should focus on identifying genetic or clinical biomarkers that predict susceptibility to irreversible motility dysfunction, ensuring safer use of GLP-1 agonists in vulnerable populations [8]. By integrating mechanistic understanding, risk stratification, and proactive monitoring, clinicians can optimize the metabolic benefits of semaglutide while minimizing its potential to disrupt gastrointestinal motility. This case is unique in that the patient developed severe gastroparesis-like symptoms and acute kidney injury after reinitiating semaglutide at a higher maintenance dose without titration, in the setting of underlying gastrointestinal comorbidities, underscoring the importance of individualized dosing and close monitoring.

## Conclusions

This case underscores the potential for semaglutide, a widely used GLP-1 receptor agonist, to induce clinically significant gastroparesis, particularly in patients with pre-existing gastrointestinal comorbidities and when proper dose titration is not followed. Early recognition of medication-induced gastroparesis is essential, as prompt discontinuation of the offending agent can lead to rapid symptom resolution and prevent serious complications such as acute kidney injury. Clinicians should maintain a high index of suspicion for drug-induced gastrointestinal side effects in patients presenting with new or worsening symptoms while on GLP-1 receptor agonists and emphasize the importance of patient education and adherence to recommended dosing protocols. As the use of semaglutide continues to expand, ongoing vigilance and individualized risk assessment are crucial to optimizing patient safety and therapeutic outcomes. This case illustrates the particular risks of restarting therapy at higher doses without titration in a patient with underlying gastrointestinal comorbidities.
